# Supplementary material for: Genome-wide association and expression quantitative trait loci studies identify multiple susceptibility loci for thyroid cancer
Source: Nat Commun. 2017 Jul 13;8:15966. doi: 10.1038/ncomms15966 (PMC5511346; doi:10.1038/ncomms15966)
Supplement: Supplementary Information [file ncomms15966-s1.pdf]

Type of file: PDF
Size of file: 0 KB
Title of file for HTML: Supplementary Information
Description: Supplementary Figures, Supplementary Tables and Supplementary References

Type of file: pdf
File size:
Title of file for HTML: Peer Review File
Description:

# Supplementary Information

Supplementary Table 1. DTC- or PTC-associated SNPs of a previous European GWAS and replicated SNPs in Asians.

| Locus | Gene | SNP | OR | *P*-value | Population | Method | References |
|---|---|---|---|---|---|---|---|
| 9q22.33 | *FOXE1* | rs965513 | 1.75 | $1.7 \times 10^{-27}$ | Iceland etc. | GWAS | (1) |
| | | | 1.65 | $4.8 \times 10^{-12}$ | Belarus | GWAS | (2) |
| | | | 1.69 | $1.3 \times 10^{-4}$ | Japanese | SNP | (3) |
| | | | 1.53 | $1.4 \times 10^{-4}$ | Chinese | SNP | (4) |
| | | | 1.59 | $4.2 \times 10^{-4}$ | Japanese | SNP | (5) |
| | | rs7028661 | 1.64 | $1.0 \times 10^{-22}$ | Spain | GWAS | (6) |
| | | rs10122541 | 1.54 | $1.1 \times 10^{-17}$ | Spain | GWAS | (6) |
| | | rs7037324 | 1.54 | $1.2 \times 10^{-17}$ | Spain | GWAS | (6) |
| 14q13.3 | *NKX2-1* | rs944289 | 1.37 | $2.0 \times 10^{-9}$ | Iceland etc. | GWAS | (1) |
| | | | 1.24 | $1.5 \times 10^{-5}$ | Spain | GWAS | (6) |
| | | | 1.21 | 0.0121 | Japanese | SNP | (3) |
| | | | 1.53 | $2.2 \times 10^{-10}$ | Chinese | SNP | (4) |
| | | | 1.23 | 0.003 | Japanese | SNP | (5) |
| | | rs116909374 | 2.09 | $4.6 \times 10^{-11}$ | Iceland etc. | GWAS | (7) |
| 2q35 | *DIRC3* | rs966423 | 1.34 | $1.3 \times 10^{-9}$ | Iceland etc. | GWAS | (7) |
| | | | 1.31 | 0.0010 | Chinese | SNP | (4) |
| | | rs6759952 | 1.21 | $6.4 \times 10^{-10}$ | Italy etc. | GWAS | (8) |
| 8p12 | *NRG1* | rs2439302 | 1.36 | $2.0 \times 10^{-9}$ | Iceland etc. | GWAS | (7) |
| | | | 1.41 | $2.78 \times 10^{-5}$ | Chinese | SNP | (4) |
| | | | 1.27 | 0.003 | Japanese | SNP | (5) |
| 7q31.1 | *IMMP2L* | rs10238549 | 1.27 | $4.1 \times 10^{-6}$ | Italy etc. | GWAS | (8) |
| | | rs7800391 | 1.25 | $5.7 \times 10^{-6}$ | Italy etc. | GWAS | (8) |
| 3q25.32 | *RARRES1* | rs7617304 | 1.25 | $4.6 \times 10^{-5}$ | Italy etc. | GWAS | (8) |
| 9q34 | *SNAPC4* | rs10781500 | 1.23 | $3.5 \times 10^{-5}$ | Italy etc. | GWAS | (8) |
| 14q24.3 | *BATF* | rs10136427 | 1.40 | $4.4 \times 10^{-7}$ | Italy etc. | GWAS | (9) |
| 20q11.23 | *DHX35* | rs7267944 | 1.39 | $2.1 \times 10^{-8}$ | Italy etc. | GWAS | (9) |
| 5q14 | *ARSB* | rs13184587 | 1.28 | $8.5 \times 10^{-6}$ | Italy etc. | GWAS | (9) |
| 13q12 | *SPATA13* | rs1220597 | 1.26 | $3.3 \times 10^{-6}$ | Italy etc. | GWAS | (9) |
| 11p15.3 | *GALNTL4* | rs7935113 | 1.36 | $7.4 \times 10^{-7}$ | Italy etc. | GWAS | (10) |
| 20p11 | *FOXA2* | rs1203952 | 1.29 | $4.4 \times 10^{-6}$ | Italy etc. | GWAS | (10) |
| 10q26.12 | *WDR11-AS1* | rs2997312 | 1.35 | $1.2 \times 10^{-4}$ | Spain | GWAS | (6) |
| | | rs10788123 | 1.26 | $5.2 \times 10^{-4}$ | Spain | GWAS | (6) |
| | | rs1254167 | 1.38 | $5.9 \times 10^{-5}$ | Spain | GWAS | (6) |
| 6q14.1 | *HTR1B* | rs4075570 | 0.82 | $2.0 \times 10^{-4}$ | Spain | GWAS | (6) |

OR, odd ratio; SNP, single nucleotide polymorphism.

Supplementary Table 2. Descriptive characteristics of the participants.

| Characteristics | Total (joint) | Stage 1 (discovery) | Stage 2 (replication) |
|---|---|---|---|
| Cases | | | |
| Number | 1085 | 470 | 615 |
| Age, years ± SD | 46.5 ± 12.1 | 43.9 ± 12.8 | 48.4 ± 11.1 |
| Male % | 14.4 % | 13.0 % | 15.4 % |
| Pathology | | | |
| PTC:FTC, *N* (%) | 997:88 (91.9:8.1) | 410:60 (87.2:12.8) | 587:28 (95.4:4.6) |
| $BRAF^{V600E}$ in PTC (%) | 186/215 (86.5) | 186/215 (86.5) | - |
| LN metastasis in PTC (%) | 419/827 (50.7) | 186/337 (55.2) | 233/490 (47.6) |
| Distant metastasis in PTC (%) | 5/769 (0.7) | 2/338 (0.6) | 3/431 (0.7) |
| ETE in PTC (%) | 519/886 (58.6) | 221/377(58.6) | 298/509 (58.5) |
| Controls | | | |
| Number | 8884 | 8279 | 605 |
| Age, years ± SD | 52.6 ± 8.8 | 52.1 ± 8.9 | 58.9 ± 4.3 |
| Male % | 47.5 % | 45.2 % | 79.2 % |

ETE, extrathyroidal extension; LN, lymph node; SD, standard deviation.

Supplementary Table 3. Forty-one candidate SNPs for stage 2 follow-up study.

| Chr | SNP | Position* | Genes | Risk alelle | Allele frequency in Cases | Allele frequency in Controls | OR | *P*-value† | SNP selection Criteria‡ |
|---|---|---|---|---|---|---|---|---|---|
| 1 | rs57075645 | 99862185 | 87kb 3' of *LPPR4* | A | 0.101 | 0.060 | 1.76 | 3.81E-07 | a |
| 1 | rs4915076 | 108359505 | Intronic *VAV3* | C | 0.233 | 0.301 | 0.71 | 9.37E-06 | a |
| 1 | rs4649295 | 233416538 | Intronic *PCNXL2* | T | 0.124 | 0.180 | 0.64 | 1.04E-05 | a |
| 2 | rs2121260 | 38268763 | intronic *FAM82A1* | G | 0.169 | 0.125 | 1.43 | 8.34E-05§ | a |
| 2 | rs1979142 | 39032703 | intronic *DHX57* | G | 0.187 | 0.132 | 1.51 | 1.76E-06 | a |
| 2 | rs1549738 | 218118722 | 30kb 3' of *DIRC3* | G | 0.388 | 0.448 | 0.78 | 2.96E-04 | c |
| 2 | rs12990503 | 218294217 | intronic *DIRC3* | G | 0.315 | 0.375 | 0.76 | 1.82E-04 | c |
| 3 | rs9858271 | 59545330 | 190kb 3' of *FHIT* | G | 0.503 | 0.426 | 1.37 | 3.57E-06 | a |
| 4 | rs1874564 | 77858105 | 13kb 5' of *SEPT11* | A | 0.233 | 0.305 | 0.69 | 3.43E-06 | a |
| 4 | rs6841841 | 182790626 | 270kb 3' of *MGC45800* | G | 0.166 | 0.232 | 0.66 | 3.68E-06 | a |
| 5 | rs10941849 | 20719433 | 731kb 5' of *CDH18* | T | 0.082 | 0.044 | 1.96 | 5.11E-08 | a |
| 5 | rs10447240 | 119328679 | 357kb 3' of *FAM170A* | A | 0.082 | 0.047 | 1.36 | 2.26E-05§ | a |
| 6 | rs16889600 | 78723162 | 550kb 5' of *HTR1B* | T | 0.556 | 0.484 | 1.83 | 9.34E-07 | c |
| 6 | rs9361385 | 78926958 | 650kb 5' of *IRAK1BP1* | C | 0.043 | 0.022 | 1.34 | 1.57E-05 | a |
| 6 | rs11754852 | 92261591 | 30kb 3' of *MIR4643* | C | 0.013 | 0.049 | 1.99 | 3.83E-05§ | a |
| 7 | rs2952745 | 52196144 | 812kb 5' of *COBL* | T | 0.372 | 0.302 | 0.25 | 4.74E-07 | a |
| 7 | rs2715152 | 82457666 | intronic *PCLO* | G | 0.069 | 0.116 | 1.37 | 6.24E-06 | a |
| 8 | rs36041430 | 24199218 | missense *ADAM28* | A | 0.324 | 0.253 | 0.57 | 1.72E-05 | a |
| 8 | rs12542743 | 32318355 | intronic *NRG1* | C | 0.300 | 0.225 | 1.42 | 1.12E-06 | a |
| 8 | rs6996585 | 32400803 | intronic *NRG1* | G | 0.261 | 0.190 | 1.48 | 1.20E-07 | a |
| 8 | rs2439302 | 32432369 | intronic *NRG1* | G | 0.269 | 0.213 | 1.36 | 8.38E-05 | b |
| 8 | rs11778356 | 55387405 | 14kb 3' of *SOX17* | A | 0.284 | 0.228 | 1.51 | 1.20E-07 | a |
| 9 | rs4628781 | 18795997 | intronic *ADAMTSL1* | C | 0.070 | 0.038 | 1.35 | 6.81E-05§ | a |
| 9 | rs10867527 | 83023162 | 682kb 3' of *TLE4* | G | 0.115 | 0.067 | 1.93 | 6.62E-07 | a |
| 9 | rs1588635 | 100537802 | 78kb 5' of *FOXE1* | A | 0.168 | 0.123 | 1.81 | 1.74E-08 | a |
| 9 | rs7028661 | 100538470 | 77kb 5' of *FOXE1* | A | 0.115 | 0.067 | 1.8 | 2.52E-08 | b |
| 9 | rs965513 | 100556109 | 59kb 5' of *FOXE1* | A | 0.109 | 0.061 | 1.91 | 2.35E-09 | b |
| 9 | rs1867277 | 100615914 | 5'-UTR *FOXE1* | A | 0.116 | 0.075 | 1.44 | 4.38E-05 | c |
| 9 | rs10122541 | 100628268 | 9kb 3' of *FOXE1* | G | 0.121 | 0.080 | 1.57 | 1.43E-05 | b |
| 9 | rs7037324 | 100658318 | 9kb 3' of *C9orf156* | A | 0.121 | 0.080 | 1.56 | 1.70E-05 | b |
| 9 | rs72753537 | 100660746 | 6kb 3' of *C9orf156* | C | 0.030 | 0.012 | 1.63 | 3.56E-06 | a |
| 11 | rs67790686 | 103885141 | Intronic *PDGFD* | C | 0.207 | 0.153 | 2.50 | 3.46E-06 | a |
| 12 | rs11175834 | 65992636 | 132kb 3' of *MSRB3* | T | 0.052 | 0.022 | 1.45 | 1.16E-05 | e |
| 12 | rs16934253 | 113737225 | 3'-UTR *SLC24A6* | A | 0.203 | 0.146 | 2.46 | 2.49E-09 | a |
| 12 | rs11061290 | 131518747 | intronic *GPR133* | T | 0.134 | 0.100 | 1.50 | 1.41E-06 | a |
| 13 | rs75150143 | 52428825 | 7.3kb 5' of *CCDC70* | C | 0.474 | 0.414 | 1.40 | 8.18E-04§ | a |
| 14 | rs34081947 | 36559531 | 208kb 3' of *MBIP* | T | 0.511 | 0.457 | 1.28 | 2.40E-04 | c |
| 14 | rs944289 | 36649246 | 119kb 3' of *MBIP* | T | 0.464 | 0.406 | 1.24 | 1.41E-03 | b |
| 14 | rs72693081 | 81453862 | Intronic *TSHR* | G | 0.429 | 0.364 | 1.27 | 4.37E-04 | d |
| 19 | rs7248104 | 7224431 | Intronic *INSR* | A | 0.114 | 0.072 | 1.31 | 6.77E-05 | d |
| 22 | rs7288885 | 26408660 | Intornic *MYO18B* | G | 0.112 | 0.075 | 1.65 | 2.42E-06 | a |

*The SNP positions are indexed to the National Center for Biotechnology Information (NCBI) build 37. ‡SNP selection criteria: a, candidate SNP, significant or suggestive association ($P < 2 \times 10^{-5}$) for our discovery stage; b, previously reported SNPs in GWASs of thyroid cancer; c-e, candidate SNPs, which showed the strongest association in regions reported by GWASs of (c) thyroid cancer and (d) TSH level[11,12] or (e) near gene related to thyroid disease[13,14]. †The *P*-value was calculated after the exclusion of relatedness using IBD. §These SNPs were included because $P < 2 \times 10^{-5}$ before IBD exclusion.

Chr, chromosome number; OR, odd ratio; SNP, single nucleotide polymorphism.

Supplementary Table 4. Comparison of the risk allele frequency between population of 1000 Genome and this study.

| Chr | SNP | Gene | Risk / Reference allele | Risk allele frequency in 1000 Genome | | | | Risk allele frequency in this study | | |
|---|---|---|---|---|---|---|---|---|---|---|
| | | | | African | American | European | East Asian | Cases | Controls | Allelic OR |
| 1 | rs4915076 | *VAV3* | T/C | 0.96 | 0.81 | 0.93 | 0.71 | 0.76 | 0.70 | 1.33 |
| 1 | rs4649295 | *PCNXL2* | C/T | 0.43 | 0.75 | 0.64 | 0.84 | 0.87 | 0.82 | 1.43 |
| 2 | rs12990503 | *DIRC3* | G/C | 0.54 | 0.35 | 0.28 | 0.60 | 0.69 | 0.63 | 1.34 |
| 2 | rs1549738 | *DIRC3* | A/G | 0.54 | 0.84 | 0.87 | 0.61 | 0.58 | 0.55 | 1.14 |
| 3 | rs9858271 | *FHIT* | G/A | 0.07 | 0.29 | 0.24 | 0.47 | 0.48 | 0.43 | 1.26 |
| 4 | rs1874564 | *SEPT11* | G/A | 0.33 | 0.52 | 0.45 | 0.66 | 0.75 | 0.69 | 1.31 |
| 8 | rs6996585 | *NRG1* | G/A | 0.24 | 0.45 | 0.42 | 0.23 | 0.29 | 0.23 | 1.39 |
| 8 | rs12542743 | *NRG1* | C/T | 0.51 | 0.56 | 0.56 | 0.26 | 0.32 | 0.25 | 1.36 |
| 8 | rs2439302 | *NRG1* | G/C | 0.47 | 0.49 | 0.48 | 0.19 | 0.27 | 0.21 | 1.37 |
| 9 | rs72753537 | *FOXE1* | C/T | 0.04 | 0.12 | 0.14 | 0.08 | 0.10 | 0.07 | 1.41 |
| 12 | rs11175834 | *MSRB3* | T/C | 0.40 | 0.10 | 0.05 | 0.14 | 0.20 | 0.15 | 1.37 |
| 12 | rs16934253 | *SLC24A6* | A/G | 0.32 | 0.08 | 0.11 | 0.01 | 0.03 | 0.02 | 1.51 |
| 14 | rs34081947 | *NKX2-1* | T/C | 0.20 | 0.43 | 0.54 | 0.39 | 0.47 | 0.41 | 1.27 |
| 14 | rs944289 | *NKX2-1* | T/C | 0.15 | 0.44 | 0.59 | 0.45 | 0.51 | 0.46 | 1.25 |
| 19 | rs7248104 | *INSR* | A/G | 0.28 | 0.40 | 0.42 | 0.32 | 0.41 | 0.36 | 1.22 |

Chr, chromosome number; OR, odd ratio; SNP, single nucleotide polymorphism.

Supplementary Table 5. Association between candidate SNPs and *cis*-eQTL results of thyroid tissues in GTEx public data.

| Chr | SNP | Position | Representative Gene | RNA-sequencing data in this study | | | Public data in normal thyroid (source: GTEx2015 v6) | |
|---|---|---|---|---|---|---|---|---|
| | | | | *Cis*-eQTL Gene | *P*-value of tumour tissue | *P*-value of normal tissue | *Cis*-eQTL Gene | *P*-value |
| 1 | rs4915076 | 108359505 | *VAV3* | *VAV3* | **0.0174** | 0.0995 | *VAV3* | **3.33E-27** |
| | | | | | | | *VAV3-AS1* | **2.02E-06** |
| 1 | rs4649295 | 233416538 | *PCNXL2* | *PCNXL2* | **0.0030** | 0.8594 | *PCNXL2* | > 0.05 |
| | | | | *NTPCR* | 0.9006 | **0.0472** | *NTPCR* | > 0.05 |
| 2 | rs1549738 | 218118722 | *DIRC3* | *TNS1* | **0.0023** | 0.1170 | *TNS1* | > 0.05 |
| 2 | rs12990503 | 218294217 | *DIRC3* | - | | | | |
| 3 | rs9858271 | 59545330 | *FHIT* | - | | | | |
| 4 | rs1874564 | 77858105 | *SEPT11* | - | | | | |
| 8 | rs6996585 | 32400803 | *NRG1* | *NRG1* | **0.0053** | 0.0526 | *NRG1* | **5.79E-21** |
| | | | | - | | | *RP11-1002K11.1* | **6.46E-19** |
| 8 | rs12542743 | 32318355 | *NRG1* | *NRG1* | **0.0073** | 0.1021 | *NRG1* | **2.50E-07** |
| | | | | - | | | *RP11-1002K11.1* | **1.00E-06** |
| 8 | rs2439302 | 32432369 | *NRG1* | *NRG1* | **0.0025** | **0.0125** | *NRG1* | **6.47E-25** |
| | | | | - | | | *RP11-1002K11.1* | **1.76E-23** |
| 9 | rs72753537 | 100660746 | *FOXE1* | *C9orf156* | 0.6914 | 0.3035 | *C9orf156* | **1.33E-05** |
| 12 | rs11175834 | 65992636 | *MSRB3* | - | | | | |
| 12 | rs16934253 | 113737225 | *SLC24A6* | - | | | | |
| 14 | rs34081947 | 36559531 | *NKX2-1* | *NKX2-1* | **0.0323** | 0.5458 | *NKX2-1* | > 0.05 |
| | | | | - | | | *RP11-116N8.4* | **2.90E-12** |
| | | | | - | | | *PTCSC3* | **1.50E-05** |
| 14 | rs944289 | 36649246 | *NKX2-1* | *NKX2-1* | **0.0069** | **0.0302** | *NKX2-1* | > 0.05 |
| | | | | - | | | *RP11-116N8.4* | **1.28E-09** |
| | | | | *SFTA3* | **0.0107** | **0.0476** | *SFTA3* | > 0.05 |
| 19 | rs7248104 | 7224431 | *INSR* | - | | | | |

The SNP positions are indexed to the National Center for Biotechnology Information (NCBI) build 37. The *cis*-eQTL gene is defined as the genes within ±500 kb the candidate SNP. The *cis*-eQTL result of candidate SNPs are from the association result of 78 tumour thyroid tissues and 23 normal thyroid tissues. The public *cis*-eQTL result of the candidate SNPs are from the GTEx (http://www.gtexportal.org). Bold indicates significance of $P < 0.05$.

Chr, chromosome number; SNP, single nucleotide polymorphism.

Supplementary Table 6. Association between candidate SNPs and the *cis*-eQTL results of various tissues other than thyroid tissue in public data.

| Chr | SNP | Position | Representative Gene | Source | eQTL-Gene | Tissue | *P*-value |
|---|---|---|---|---|---|---|---|
| 1 | rs4915076 | 108359505 | *VAV3* | Westra2013 | *VAV3* | Whole blood | 1.56E-08 |
| | | | | GTEx2015_v6 | *VAV3* | Lung | 3.23E-08 |
| | | | | GTEx2015_v6 | *VAV3* | Whole blood | 1.91E-06 |
| 1 | rs4649295 | 233416538 | *PCNXL2* | - | - | - | - |
| 2 | rs12990503 | 218294217 | *DIRC3* | GTEx2015_v6 | *DIRC3* | Skin | 7.19E-06 |
| 2 | rs1549738 | 218118722 | *DIRC3* | - | - | - | - |
| 3 | rs9858271 | 59545330 | *FHIT* | - | - | - | - |
| 4 | rs1874564 | 77858105 | *SEPT11* | Westra2013 | *CCNI* | Whole blood | 0.0013 |
| | | | | Westra2013 | *SEPT11* | Whole blood | 0.0018 |
| 8 | rs12542743 | 32318355 | *NRG1* | - | | | |
| 8 | rs6996585 | 32400803 | *NRG1* | Westra2013 | *NRG1* | Whole blood | 3.95E-190 |
| | | | | GTEx2015_v6 | *NRG1* | Whole blood | 2.49E-11 |
| | | | | GTEx2015_v6 | *RP11-1002K11.1* | Whole blood | 7.74E-08 |
| 8 | rs2439302 | 32432369 | *NRG1* | Westra2013 | *NRG1* | Whole blood | 9.81E-198 |
| | | | | GTEx2015_v6 | *NRG1* | Whole blood | 1.68E-13 |
| | | | | GTEx2015_v6 | *RP11-1002K11.1* | Whole blood | 1.68E-09 |
| 9 | rs72753537 | 100660746 | *FOXE1* | GTEx2015_v6 | *C9orf156* | Adipose | 1.15E-05 |
| | | | | GTEx2015_v6 | *C9orf156* | Skeletal muscle | 3.79E-06 |
| | | | | GTEx2015_v6 | *C9orf156* | Nerve, Tibia | 5.04E-06 |
| | | | | GTEx2015_v6 | *C9orf156* | Testis | 1.67E-05 |
| 12 | rs11175834 | 65992636 | *MSRB3* | - | - | - | - |
| 12 | rs16934253 | 113737225 | *SLC24A6* | Westra2013 | *AC010178.40-2* | Whole blood | 3.50E-04 |
| | | | | Westra2013 | *C12orf52* | Whole blood | 0.0029 |
| | | | | Westra2013 | *SLC24A6* | Whole blood | 3.67E-26 |
| 14 | rs34081947 | 36559531 | *NKX2-1* | GTEx2015_v6 | *RP11-116N8.4* | Adipose | 2.90E-07 |
| 14 | rs944289 | 36649246 | *NKX2-1* | GTEx2015_v6 | *RP11-116N8.4* | Adipose | 5.79E-06 |
| 19 | rs7248104 | 7224431 | *INSR* | GTEx2015_v6 | *INSR* | Nerve, Tibia | 2.70E-06 |

The SNP positions are indexed to the National Center for Biotechnology Information (NCBI) build 37. The *cis*-eQTL result of candidate SNPs are from the GTEx (http://www.gtexportal.org) and Whole blood eQTL (Westra 2013).

Chr, chromosome number; SNP, single nucleotide polymorphism.

Supplementary Table 7. Gene set enrichment analysis results for the candidate SNPs

| Chr | SNP | Gene | Normal thyroid tissue | | Tumour thyroid tissue | | Significant gene set list |
|---|---|---|---|---|---|---|---|
| | | | *N* of gene set (FDR *q*<0.05) | Lowest FDR *q* | *N* of gene set (FDR *q*<0.05) | Lowest FDR *q* | |
| 1 | rs4649295 | *PCNXL2* | 0 | 0.662 | 0 | 0.160 | - |
| 1 | rs4915076 | *VAV3* | 0 | 0.108 | **2** | **0.026** | (KEGG) Steroid Hormone Biosynthesis, (Reactome) Steroid Hormones |
| 2 | rs12990503 | *DIRC3* | 0 | 0.685 | **1** | **0.038** | (Reactome) TGF beta receptor signaling activates SMADS |
| 2 | rs1549738 | *DIRC3* | 0 | 0.267 | 0 | 0.200 | - |
| 3 | rs9858271 | *FHIT* | 0 | 0.192 | 0 | 0.569 | - |
| 4 | rs1874564 | *SEPT11* | 0 | 0.499 | **1** | **0.042** | (Biocarta) ATM Pathway |
| 8 | rs12542743 | *NRG1* | 0 | 0.234 | 0 | 0.860 | - |
| 8 | rs6996585 | *NRG1* | **31** | **< 0.001** | 0 | 0.936 | (Biocarta) AT1R Pathway, CXCR4 Pathway, EIF4 Pathway, FCER1 Pathway, FMLP Pathway, GH Pathway, GLEEVEC Pathway, GPCR Pathway, GSK3 Pathway, HCMV Pathway, IGF1 Pathway, IL6 Pathway, Insulin Pathway, MEF2D Pathway, MET Pathway, NFAT Pathway, NFkB Pathway, NGF Pathway, PDGF Pathway, PYK2 Pathway, Stress Pathway, TCR Pathway, VEGF Pathway<br>(KEGG) Axon guidance, Colorectal Cancer<br>(Reactome) Downstream signal trransduction, NGF signalling via TRKA from the plasma membrane, Regulation of KIT signaling, Signaling by FGFR, Signaling by NGF, Transcriptional regulaiton of white adipocyte differntiation |
| 8 | rs2439302 | *NRG1* | 0 | 0.074 | 0 | 0.761 | - |
| 9 | rs72753537 | *FOXE1* | 0 | 0.152 | 0 | 0.400 | - |
| 12 | rs11175834 | *MSRB3* | 0 | 0.686 | 0 | 0.810 | - |
| 12 | rs16934253 | *SLC24A6* | NA | NA | 0 | 0.997 | - |
| 14 | rs34081947 | *NKX2-1* | 0 | 0.110 | 0 | 0.529 | - |
| 14 | rs944289 | *NKX2-1* | 0 | 0.803 | 0 | 0.059 | - |
| 19 | rs7248104 | *INSR* | **1** | **0.028** | 0 | 0.650 | (Biocarta) ERK Pathway |

1077 gene sets (BioCarta, KEGG and Reactome) of Molecular Signatures Database (MSigDB version 5.1) were used. Bold indicates significance of FDR $q < 0.05$.

Chr, chromosome number; FDR *q*, false discovery rate *q*-value; OR, odd ratio; SNP, single nucleotide polymorphism.

Supplementary Table 8. Significantly enriched gene sets (FDR $q < 0.05$) according to the rs6996585 genotype.

| Gene set | Description | NES | Nor *P* | FDR *q* |
|---|---|---|---|---|
| (Biocarta) AT1R Pathway | Angiotensin II mediated activation of JNK Pathway via Pyk2 dependent signaling | 2.316 | < 0.001 | 0.001 |
| (Biocarta) PYK2 Pathway | Links between Pyk2 and Map Kinases | 2.273 | < 0.001 | 0.004 |
| (Biocarta) IGF1 Pathway | IGF-1 Signaling Pathway | 2.108 | < 0.001 | 0.018 |
| (Biocarta) Insulin Pathway | Insulin Signaling Pathway | 2.044 | < 0.001 | 0.02 |
| (Biocarta) MET Pathway | Signaling of Hepatocyte Growth Factor Receptor | 2.054 | < 0.001 | 0.02 |
| (Reactome) Transcriptional regulation of white adipocyte differentiation | Genes involved in Transcriptional Regulation of White Adipocyte Differentiation | 2.124 | < 0.001 | 0.021 |
| (Biocarta) NGF Pathway | Nerve growth factor pathway | 2.06 | < 0.001 | 0.022 |
| (Biocarta) GLEEVEC Pathway | Inhibition of Cellular Proliferation by Gleevec | 2.084 | < 0.001 | 0.022 |
| (Biocarta) GSK3 Pathway | Inactivation of Gsk3 by AKT causes accumulation of b-catenin in Alveolar Macrophages | 2.022 | 0.002 | 0.024 |
| (Biocarta) PDGF Pathway | PDGF Signaling Pathway | 2.026 | < 0.001 | 0.024 |
| (Biocarta) FCER1 Pathway | Fc Epsilon Receptor I Signaling in Mast Cells | 2.002 | < 0.001 | 0.029 |
| (Reactome) NGF signaling via TRKA | Genes involved in NGF signaling via TRKA from the plasma membrane | 1.968 | < 0.001 | 0.041 |
| (Biocarta) IL6 Pathway | IL 6 signaling pathway | 1.93 | < 0.001 | 0.043 |
| (Biocarta) GPCR Pathway | Signaling Pathway from G-Protein Families | 1.931 | < 0.001 | 0.044 |
| (Reactome) Downstream signal transduction | Genes involved in Downstream signal transduction | 1.936 | < 0.001 | 0.044 |
| (Biocarta) CXCR4 Pathway | CXCR4 Signaling Pathway | 1.95 | < 0.001 | 0.045 |
| (Biocarta) MEF2D Pathway | Role of MEF2D in T-cell Apoptosis | 1.938 | < 0.001 | 0.045 |
| (Biocarta) NFkB Pathway | NF-kB Signaling Pathway | 1.941 | < 0.001 | 0.046 |
| (KEGG) Colorectal Cancer | Colorectal cancer | 1.826 | 0.002 | 0.048 |
| (Biocarta) EIF4 Pathway | Regulation of eIF4e and p70 S6 Kinase | 1.837 | < 0.001 | 0.048 |
| (Biocarta) HCMV Pathway | Human Cytomegalovirus and Map Kinase Pathways | 1.813 | 0.002 | 0.048 |
| (KEGG) Axon guidance | Axon guidance | 1.839 | 0.008 | 0.048 |
| (Biocarta) Stress Pathway | TNF/Stress Related Signaling | 1.822 | 0.008 | 0.048 |
| (Biocarta) GH Pathway | Growth Hormone Signaling Pathway | 1.951 | 0.008 | 0.048 |
| (Biocarta) NFAT Pathway | NFAT and Hypertrophy of the heart (Transcription in the broken heart) | 1.912 | < 0.001 | 0.049 |
| (Biocarta) TCR Pathway | T Cell Receptor Signaling Pathway | 1.837 | < 0.001 | 0.049 |
| (Reactome) Signaling by FGFR | FGFR Signaling pathway | 1.816 | 0.004 | 0.049 |
| (Reactome) Signaling by NGF | Genes involved in Signaling by NGF | 1.839 | 0.002 | 0.049 |
| (Biocarta) FMLP Pathway | fMLP induced chemokine gene expression in HMC-1 cells | 1.828 | 0.004 | 0.049 |
| (Biocarta) VEGF Pathway | VEGF, Hypoxia, and Angiogenesis | 1.833 | < 0.001 | 0.049 |
| (Reactome) Regulation of KIT signaling | Genes involved in Regulation of KIT signaling | 1.817 | 0.004 | 0.049 |

1077 gene sets (BioCarta, KEGG and Reactome) of Molecular Signatures Database (MSigDB version 5.1) were used.

FDR *q*, false discovery rate *q*-value; NES, normalized enrichment score; Nor *P*, nominal *P*-value.

Supplementary Table 9. Associations between the candidate SNPs and clinical phenotypes.

| Chr | SNP | Gene | Clinical phenotype | Genotypes | | | Total | *P*-value | *BRAF* positive | *BRAF* negative |
|---|---|---|---|---|---|---|---|---|---|---|
| | rs4915076 | *VAV3* | | CC | C**T** | **TT** | | | | |
| | | | *BRAF*$^{V600E}$ | 10/11(90.9%) | 73/83(88.0%) | 103/121(85.1%) | 186/215(86.5%) | 0.477 | | |
| | | | LN metastasis | 11/17(64.7%) | 71/124(57.3%) | 104/196(53.1%) | 186/337(55.2%) | 0.289 | 0.855 | 0.653 |
| | | | ETE | 10/20(50.0%) | 78/134(58.2%) | 133/223(59.6%) | 221/377(58.6%) | 0.483 | 0.634 | 0.573 |
| 1 | rs4649295 | *PCNXL2* | | **CC** | **C**T | TT | | | | |
| | | | *BRAF*$^{V600E}$ | 141/162(87.0%) | 40/46(87.0%) | 4/6(66.7%) | 185/214(86.4%) | 0.377 | | |
| | | | LN metastasis | 145/256(56.6%) | 34/72(47.2%) | 3/4(75.0%) | 182/332(54.8%) | 0.356 | 0.930 | 0.765 |
| | | | ETE | 169/285(59.3%) | 46/80(57.5%) | 3/7(42.9%) | 218/372(58.6%) | 0.489 | 0.302 | 0.107 |
| 2 | rs12990503 | *DIRC3* | | **CC** | **C**G | GG | | | | |
| | | | *BRAF*$^{V600E}$ | 94/105(89.5%) | 71/87(81.6%) | 20/22(90.9%) | 185/214(86.4%) | 0.491 | | |
| | | | LN metastasis | 94/162(58.0%) | 69/137(50.4%) | 23/37(62.2%) | 186/336(55.4%) | 0.763 | 0.928 | 0.041 |
| | | | ETE | 108/183(59.0%) | 85/153(55.6%) | 27/40(67.5%) | 220/376(58.5%) | 0.669 | 0.584 | 0.290 |
| 2 | rs1549738 | *DIRC3* | | **AA** | **A**G | GG | | | | |
| | | | *BRAF*$^{V600E}$ | 65/74(87.8%) | 98/113(86.7%) | 21/26(80.8%) | 184/213(86.4%) | 0.462 | | |
| | | | LN metastasis | 65/122(53.3%) | 89/161(55.3%) | 31/52(59.6%) | 185/335(55.2%) | 0.459 | 0.441 | 0.879 |
| | | | ETE | 82/136(60.3%) | 111/184(60.3%) | 26/55(47.3%) | 219/375(58.4%) | 0.187 | 0.618 | 0.877 |
| 3 | rs9858271 | *FHIT* | | AA | A**G** | **GG** | | | | |
| | | | *BRAF*$^{V600E}$ | 35/45(77.8%) | 101/111(91.1%) | 47/56(83.9%) | 183/212(86.3%) | 0.478 | | |
| | | | LN metastasis | 42/74(56.8%) | 98/175(56.0%) | 42/82(51.2%) | 182/331(55.0%) | 0.492 | 0.328 | 0.424 |
| | | | ETE | 49/81(60.5%) | 114/191(59.7%) | 55/99(55.6%) | 218/371(58.8%) | 0.494 | 0.934 | 0.105 |
| 4 | rs1874564 | *SEPT11* | | AA | A**G** | **GG** | | | | |
| | | | *BRAF*$^{V600E}$ | 11/12(91.7%) | 68/76(89.5%) | 106/126(84.1%) | 185/214(86.4%) | 0.236 | | |
| | | | LN metastasis | 13/20(65.0%) | 67/118(56.8%) | 106/196(54.1%) | 186/334(55.7%) | 0.358 | 0.229 | 0.381 |
| | | | ETE | 11/22(50.0%) | 75/130(57.7%) | 134/223(60.1%) | 220/375(58.7%) | 0.372 | 0.688 | **0.009** |
| 8 | rs12542743 | *NRG1* | | **CC** | **C**T | TT | | | | |
| | | | *BRAF*$^{V600E}$ | 23/27(85.2%) | 89/103(86.4%) | 72/83(86.7%) | 184/213(86.4%) | 0.855 | | |
| | | | LN metastasis | 27/38(71.1%) | 83/155(53.5%) | 75/140(53.6%) | 185/333(55.6%) | 0.156 | **0.025** | 0.545 |
| | | | ETE | 26/43(60.5%) | 110/173(63.6%) | 82/157(52.2%) | 218/373(58.4%) | 0.097 | 0.152 | 0.752 |
| 8 | rs6996585 | *NRG1* | | AA | A**G** | **GG** | | | | |
| | | | *BRAF*$^{V600E}$ | 80/92(87.0%) | 81/92(88.0%) | 24/29(82.8%) | 185/213(86.9%) | 0.700 | | |
| | | | LN metastasis | 85/153(55.6%) | 72/146(49.3%) | 26/35(74.3%) | 183/334(54.8%) | 0.348 | **0.015** | 0.585 |
| | | | ETE | 96/170(56.5%) | 97/166(58.4%) | 24/37(64.9%) | 217/373(58.2%) | 0.393 | 0.067 | 0.377 |
| 8 | rs2439302 | *NRG1* | | CC | C**G** | **GG** | | | | |
| | | | *BRAF*$^{V600E}$ | 84/96(87.5%) | 71/85(83.5%) | 18/21(85.7%) | 173/202(85.6%) | 0.589 | | |
| | | | LN metastasis | 87/159(54.7%) | 65/134(48.5%) | 19/23(82.6%) | 171/316(54.1%) | 0.322 | **0.022** | 0.908 |
| | | | ETE | 99/175(56.6%) | 88/153(57.5%) | 17/25(68.0%) | 204/353(57.8%) | 0.430 | 0.107 | 0.376 |
| 9 | rs72753537 | *FOXE1* | | **CC** | **C**T | TT | | | | |
| | | | *BRAF*$^{V600E}$ | 5/6(83.3%) | 44/48(91.7%) | 136/160(85.0%) | 185/214(86.4%) | 0.386 | | |
| | | | LN metastasis | 6/8(75.0%) | 35/68(51.5%) | 145/260(55.8%) | 186/336(55.4%) | 0.907 | 0.460 | 0.636 |
| | | | ETE | 4/9(44.4%) | 44/77(57.1%) | 173/290(59.7%) | 221/376(58.8%) | 0.392 | 0.566 | 0.212 |
| 12 | rs11175834 | *MSRB3* | | CC | C**T** | **TT** | | | | |
| | | | *BRAF*$^{V600E}$ | 116/132(87.9%) | 56/67(83.6%) | 10/12(83.3%) | 182/211(86.3%) | 0.392 | | |
| | | | LN metastasis | 123/218(56.4%) | 48/95(50.5%) | 11/18(61.1%) | 182/331(55.0%) | 0.691 | 0.403 | 0.746 |
| | | | ETE | 145/242(59.9%) | 61/108(56.5%) | 10/18(55.6%) | 216/368(58.7%) | 0.506 | 0.600 | 0.533 |
| 12 | rs16934253 | *SLC24A6* | | **AA** | **A**G | GG | | | | |
| | | | *BRAF*$^{V600E}$ | 0/0 (0%) | 22/25(88.0%) | 164/190(86.3%) | 186/215(86.5%) | 0.822 | | |
| | | | LN metastasis | 0/0 (0%) | 17/37(45.9%) | 169/300(56.3%) | 186/337(55.2%) | 0.245 | 0.064 | 0.127 |
| | | | ETE | 0/0 (0%) | 23/39(59.0%) | 198/338(58.6%) | 221/377(58.6%) | 0.963 | 0.592 | 0.392 |
| 14 | rs34081947 | *NKX2-1* | | CC | C**T** | **TT** | | | | |
| | | | *BRAF*$^{V600E}$ | 59/63(93.7%) | 91/107(85.0%) | 35/43(81.4%) | 185/213(86.9%) | 0.056 | | |
| | | | LN metastasis | 50/92(54.3%) | 94/172(54.7%) | 42/72(58.3%) | 186/336(55.4%) | 0.633 | 0.509 | 0.726 |
| | | | ETE | 58/102(56.9%) | 119/193(61.7%) | 43/80(53.8%) | 220/375(58.7%) | 0.756 | 0.945 | 0.836 |
| 14 | rs944289 | *NKX2-1* | | CC | C**T** | **TT** | | | | |
| | | | *BRAF*$^{V600E}$ | 52/58(89.7%) | 89/104(85.6%) | 45/53(84.9%) | 186/215(86.5%) | 0.450 | | |
| | | | LN metastasis | 43/84(51.2%) | 91/167(54.5%) | 52/86(60.5%) | 186/337(55.2%) | 0.221 | 0.206 | 0.634 |
| | | | ETE | 53/92(57.6%) | 112/192(58.3%) | 56/93(60.2%) | 221/377(58.6%) | 0.721 | 0.338 | 0.758 |
| 19 | rs7248104 | *INSR* | | **AA** | **A**G | GG | | | | |
| | | | *BRAF*$^{V600E}$ | 34/39(87.2%) | 86/98(87.8%) | 60/71(84.5%) | 180/208(86.5%) | 0.623 | | |

| | | | | | | | | |
|---|---|---|---|---|---|---|---|---|
| | LN metastasis | 30/57(52.6%) | 87/164(53.0%) | 63/108(58.3%) | 180/329(54.7%) | 0.419 | 0.355 | 0.156 |
| | ETE | 49/70(70.0%) | 107/181(59.1%) | 59/116(50.9%) | 215/367(58.6%) | **0.010** | 0.571 | **0.001** |

All analyses were conducted with papillary thyroid cancer. Bold genotype indicates risk allele. Bold *P*-value indicates significance of < 0.05.
Chr, chromosome number; ETE, extrathyroidal extension; LN, lymph node; OR, odd ratio; SNP, single nucleotide polymorphism.

Supplementary Table 10. SNPs in the *FOXE1* region and DTC and PTC associations in a Korean population.

| Chr | SNP | Position | Gene | Risk Allele | Stage | DTC | | | PTC | | |
|---|---|---|---|---|---|---|---|---|---|---|---|
| | | | | | | Allele Frequency (case/control) | OR | *P* | Allele Frequency (case/control) | OR | *P* |
| 9 | rs965513 | 100556109 | *FOXE1* | A | Discovery | 0.11/0.06 | 1.91 | 2.35E-09 | 0.11/0.06 | 1.94 | 8.20E-09 |
| | | | | | Replication | 0.09/0.07 | 1.25 | 0.1372 | 0.09/0.07 | 1.23 | 0.1653 |
| | | | | | Joint | 0.10/0.06 | 1.67 | 6.20E-11 | 0.10/0.06 | 1.66 | 4.48E-10 |
| 9 | rs1588635 | 100537802 | *FOXE1* | A | Discovery | 0.11/0.07 | 1.81 | 1.74E-08 | 0.12/0.07 | 1.83 | 6.76E-08 |
| | | | | | Replication | 0.09/0.08 | 1.18 | 0.2425 | 0.09/0.08 | 1.17 | 0.2706 |
| | | | | | Joint | 0.10/0.07 | 1.58 | 2.36E-09 | 0.10/0.07 | 1.57 | 1.30E-08 |
| 9 | rs7028661 | 100538470 | *FOXE1* | A | Discovery | 0.11/0.07 | 1.80 | 2.52E-08 | 0.12/0.07 | 1.82 | 9.47E-08 |
| | | | | | Replication | 0.09/0.08 | 1.20 | 0.2090 | 0.09/0.08 | 1.19 | 0.2343 |
| | | | | | Joint | 0.10/0.07 | 1.57 | 3.08E-09 | 0.10/0.07 | 1.56 | 1.64E-08 |
| 9 | rs10122541 | 100628268 | *FOXE1* | G | Discovery | 0.12/0.08 | 1.57 | 1.43E-05 | 0.10/0.06 | 1.81 | 4.07E-07 |
| | | | | | Replication | 0.10/0.08 | 1.22 | 0.1690 | 0.08/0.07 | 1.03 | 0.8341 |
| | | | | | Joint | 0.11/0.08 | 1.36 | 4.25E-06 | 0.09/0.06 | 1.48 | 4.77E-06 |
| 9 | rs72753537 | 100660746 | *FOXE1* | C | Discovery | 0.12/0.07 | 1.63 | 3.56E-06 | 0.12/0.07 | 1.76 | 1.70E-07 |
| | | | | | Replication | 0.09/0.07 | 1.38 | **0.0352** | 0.09/0.07 | 1.43 | **0.0209** |
| | | | | | Joint | 0.10/0.07 | 1.41 | 7.67E-06 | 0.11/0.07 | 1.48 | 5.37E-07 |
| 9 | rs7037324 | 100658318 | *FOXE1* | A | Discovery | 0.12/0.08 | 1.56 | 1.70E-05 | 0.19/0.13 | 1.56 | 1.43E-06 |
| | | | | | Replication | 0.10/0.08 | 1.28 | 0.0865 | 0.15/0.14 | 1.11 | 0.3758 |
| | | | | | Joint | 0.11/0.08 | 1.38 | 1.28E-05 | 0.17/0.13 | 1.32 | 1.62E-05 |
| 9 | rs1867277 | 100615914 | *FOXE1* | A | Discovery | 0.17/0.12 | 1.44 | 4.38E-05 | 0.18/0.12 | 1.54 | 4.96E-06 |
| | | | | | Replication | 0.12/0.13 | 0.89 | 0.3109 | 0.11/0.13 | 0.86 | 0.1982 |
| | | | | | Joint | 0.14/0.12 | 1.18 | 0.0176 | 0.15/0.12 | 1.19 | 0.0146 |

The SNP positions are indexed to the National Center for Biotechnology Information (NCBI) build 37. Bold indicates significance of $P < 0.05$ in replication stage.

Chr, chromosome number; OR, odd ratio; SNP, single nucleotide polymorphism.

Supplementary Table 11. A comparison of previously reported SNPs associated with DTC or PTC in Europeans and Koreans.

| Gene | SNP | Europeans | | | DTC in Koreans | | PTC in Koreans | |
|---|---|---|---|---|---|---|---|---|
| | | OR | *P*-value | population | OR | *P*-value | OR | *P*-value |
| *FOXE1* | rs965513 | 1.75 | $1.7 \times 10^{-27}$ | Iceland etc. | 1.91 | $2.4 \times 10^{-9}$ | 1.94 | $8.2 \times 10^{-9}$ |
| *FOXE1* | rs7028661 | 1.64 | $1.0 \times 10^{-22}$ | Spain | 1.80 | $2.5 \times 10^{-8}$ | 1.82 | $9.5 \times 10^{-8}$ |
| *FOXE1* | rs10122541 | 1.54 | $1.1 \times 10^{-17}$ | Spain | 1.57 | $1.4 \times 10^{-5}$ | 1.69 | $1.2 \times 10^{-6}$ |
| *FOXE1* | rs7037324 | 1.54 | $1.2 \times 10^{-17}$ | Spain | 1.56 | $1.7 \times 10^{-5}$ | 1.68 | $1.4 \times 10^{-6}$ |
| *NRG1* | rs2439302 | 1.36 | $2.0 \times 10^{-9}$ | Iceland etc. | 1.34 | $8.4 \times 10^{-5}$ | 1.48 | $1.5 \times 10^{-6}$ |
| *NKX2-1* | rs944289 | 1.37 | $2.0 \times 10^{-9}$ | Iceland etc. | 1.24 | 0.0014 | 1.22 | 0.0062 |
| *DIRC3* | rs966423 | 1.34 | $1.3 \times 10^{-9}$ | Iceland etc. | 1.24 | 0.0081 | 1.27 | 0.0067 |
| *DIRC3* | rs6759952 | 1.21 | $6.4 \times 10^{-10}$ | Italy etc. | 1.21 | 0.0164 | 1.25 | 0.0107 |
| *IMMP2L* | rs10238549 | 1.27 | $4.1 \times 10^{-6}$ | Italy etc. | 1.10 | 0.3542 | 1.17 | 0.1343 |
| *IMMP2L* | rs7800391 | 1.25 | $5.7 \times 10^{-6}$ | Italy etc. | 1.03 | 0.7271 | 1.00 | 0.9850 |
| *DHX35* | rs7267944 | 1.39 | $2.1 \times 10^{-8}$ | Italy etc. | 0.98 | 0.8209 | 0.98 | 0.7752 |
| *ARSB* | rs13184587 | 1.28 | $8.5 \times 10^{-6}$ | Italy etc. | 1.03 | 0.7453 | 1.02 | 0.8216 |
| *WDR11-AS1* | rs2997312 | 1.35 | $1.2 \times 10^{-4}$ | Spain | 0.94 | 0.6299 | 0.97 | 0.8039 |
| *WDR11-AS1* | rs10788123 | 1.26 | $5.2 \times 10^{-4}$ | Spain | 0.92 | 0.2401 | 0.94 | 0.4540 |

OR, odd ratio; SNP, single nucleotide polymorphism.

Supplementary Table 12. Variants of six recently reported novel and replicating loci for thyroid cancer in a Korean population.

| Locus | SNP | Position | Annotation/ Nearby gene(s) | *P*-value of Gudmundsson et al. | Discovery result in this study | | |
|---|---|---|---|---|---|---|---|
| | | | | | *P*-value | Nearby top SNP with *P* < 0.001 | *P*-value (Joint *P*) of nearby top SNP |
| 1q42.2 | rs12129938 | 233,276,815 | Intron variant *PCNXL2* | 4.0E-11 | 0.002 | rs4649295 | 1.04E-05 (6.00E-08) |
| 10q24.33 | rs7902587 | 103,934,543 | Intergenic variant *OBFC1* | 5.4E-11 | NA | rs4244255 | 8.72E-06 |
| 5q22.1 | rs73227498 | 112,150,207 | Intergenic variant *NREP, EPB41L4A* | 3.0E-10 | NA | - | |
| 15q22.33 | rs2289261 | 67,165,147 | Intron variant *SMAD3* | 3.1E-9 | 0.2844 | - | |
| 3q26.2 | rs6793295 | 169,800,667 | Missense variant *TERC, LRRC34* | 2.7E-8 | 0.0474 | - | |
| 5p15.33 | rs10069690 | 1,279,675 | Intron variant *TERT* | 3.2E-7 | NA | - | |

NA, not available; SNP, single nucleotide polymorphism.

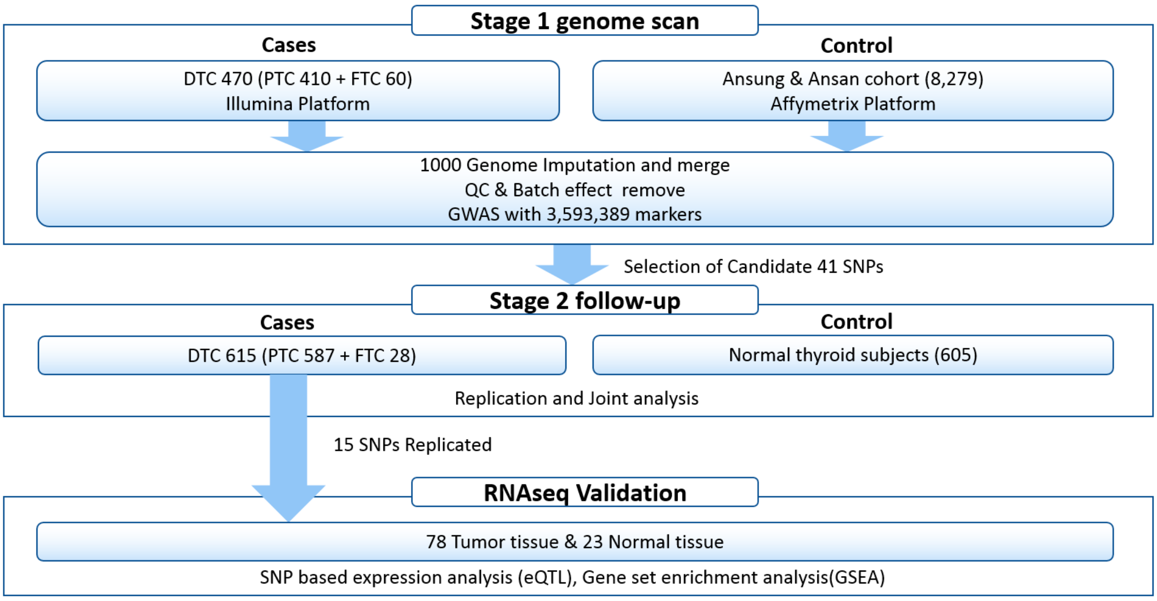


**Supplementary Figure 1. Overview of the study flow.** The number of individuals with DTC (PTC + FTC) or unaffected individual; the imputed or replicated genotypes are shown for each study flow.

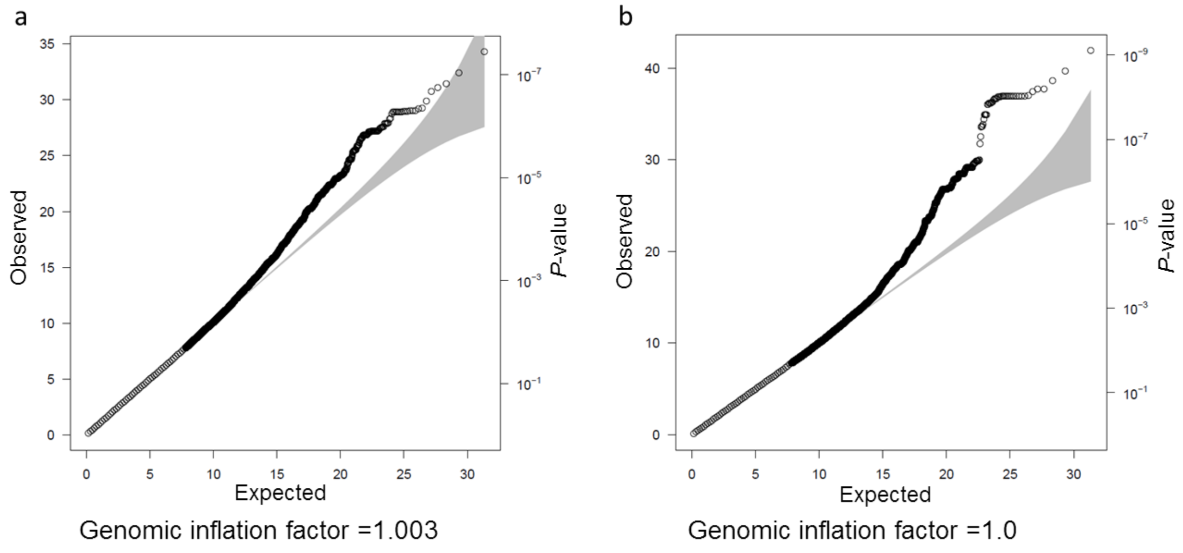


**Supplementary Figure 2. Quantile-quantile plot for stage 1 genome scans.** A quantile-quantile plot for (a) DTC and (b) PTC showing the distribution of the observed *P*-values from association testing in the stage 1 genome scan against the expected distribution under the null hypothesis. The grey zone indicates the 95% confidence interval.

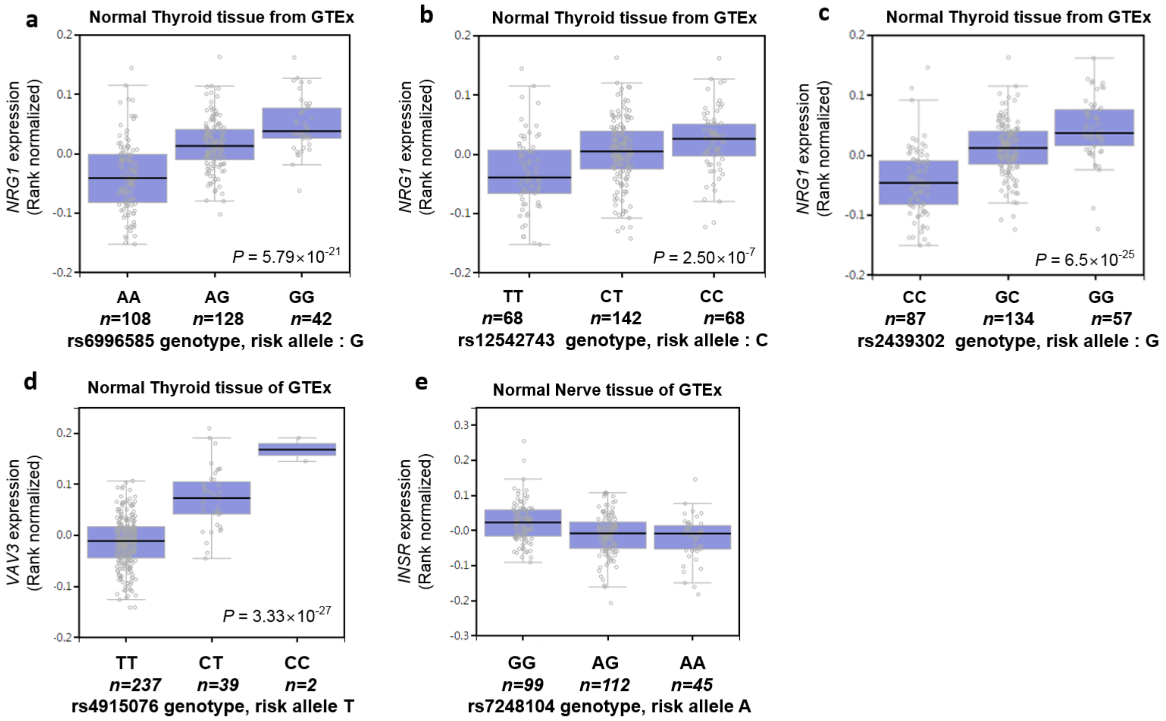


**Supplementary Figure 3. *Cis*-eQTL results from GTEx public data.** The *cis*-eQTL results of *NRG1* in normal thyroid tissue according to the (a) rs6996585, (b) rs12542743 and (c) rs2439302 genotypes. The *cis*-eQTL results of (d) *VAV3* in normal thyroid tissue according to the rs4915076 genotype and (e) *INSR* in normal nerve tissue according to the rs7248104 genotype from GTEx2015 v6 data (http://www.gtexportal.org).

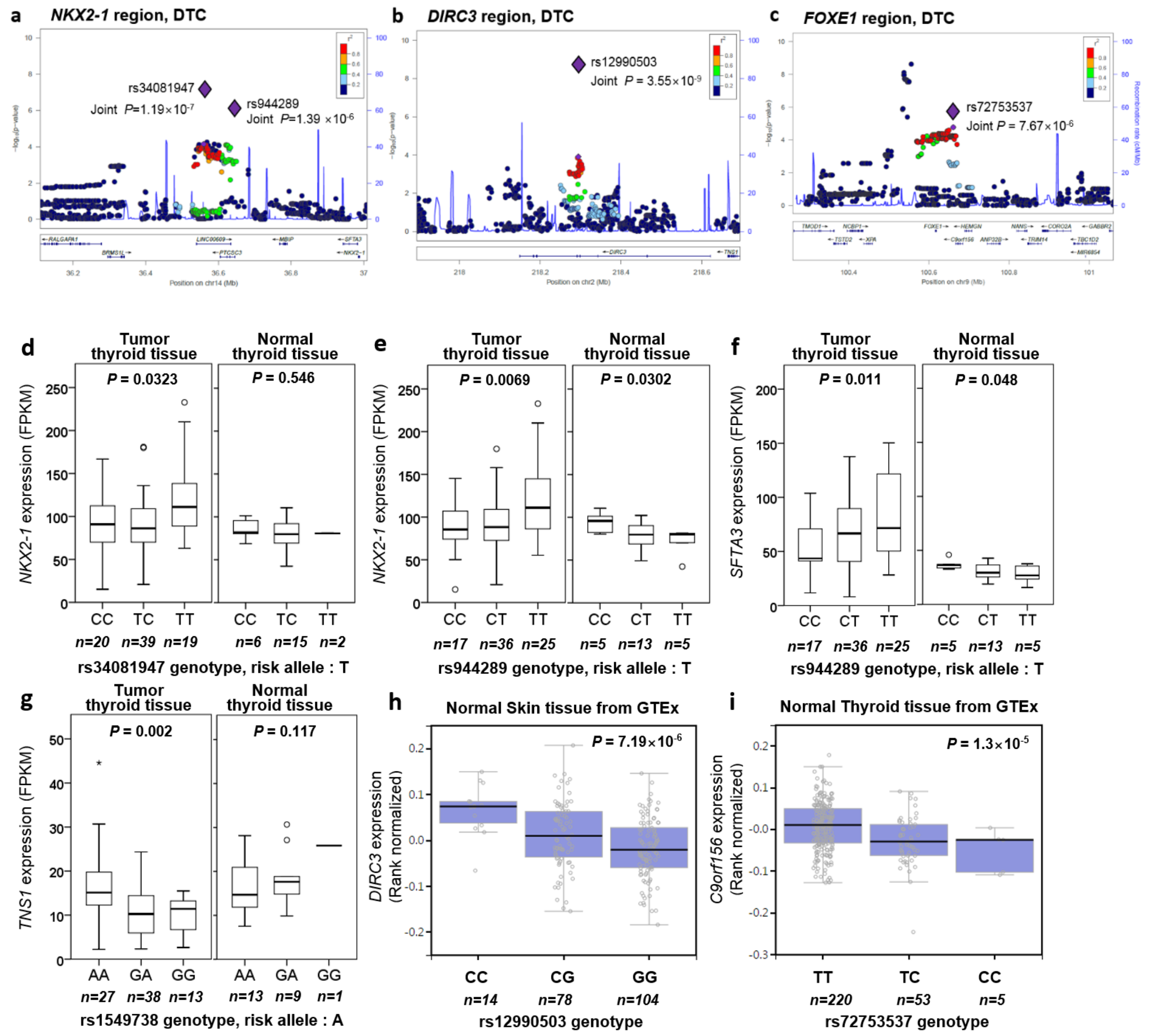


**Supplementary Figure 4. Regional association plots and expression for previously documented loci.** A regional association plot for (a) *NKX2-1*, (b) *DIRC3* and (c) *FOXE1*. The large purple diamonds indicate the most or second most associated SNP according to joint analyses and nearby SNPs are colour coded according to the level of LD with the top SNP. The left y-axis shows the significance of the association on a $-\log_{10}$ scale, and the right y-axis shows a recombination rate across the region. Estimated recombination rates from the 1000 Genomes ASN, hg19 database are plotted with the blue line to reflect the local LD structure. The *cis*-eQTL results of *NKX2-1* according to the (d) rs34081947 and (e) rs944289 genotypes; (f) *SFTA3* according to the rs944289 genotypes; and (g) *TNS1* according to the rs1549738 genotypes in tumour and normal thyroid tissues. Error bars represent s.e.m. The *cis*-eQTL results of (h) *DIRC3* in skin tissue and (i) *C9orf156* in thyroid tissue from GTEx2015 v6 data (http://www.gtexportal.org) according to the rs12990503 and rs72753537 genotypes, respectively.

## Supplementary Reference


1. Gudmundsson, J. *et al.* Common variants on 9q22.33 and 14q13.3 predispose to thyroid cancer in European populations. *Nat Genet* **41**, 460-4 (2009).
2. Takahashi, M. *et al.* The FOXE1 locus is a major genetic determinant for radiation-related thyroid carcinoma in Chernobyl. *Hum Mol Genet* **19**, 2516-23 (2010).
3. Matsuse, M. *et al.* The FOXE1 and NKX2-1 loci are associated with susceptibility to papillary thyroid carcinoma in the Japanese population. *J Med Genet* **48**, 645-8 (2011).
4. Wang, Y.L. *et al.* Confirmation of papillary thyroid cancer susceptibility loci identified by genome-wide association studies of chromosomes 14q13, 9q22, 2q35 and 8p12 in a Chinese population. *J Med Genet* **50**, 689-95 (2013).
5. Rogounovitch, T.I. *et al.* The common genetic variant rs944289 on chromosome 14q13.3 associates with risk of both malignant and benign thyroid tumors in the Japanese population. *Thyroid* **25**, 333-40 (2015).
6. Mancikova, V. *et al.* Thyroid cancer GWAS identifies 10q26.12 and 6q14.1 as novel susceptibility loci and reveals genetic heterogeneity among populations. *Int J Cancer* **137**, 1870-8 (2015).
7. Gudmundsson, J. *et al.* Discovery of common variants associated with low TSH levels and thyroid cancer risk. *Nat Genet* **44**, 319-22 (2012).
8. Kohler, A. *et al.* Genome-wide association study on differentiated thyroid cancer. *J Clin Endocrinol Metab* **98**, E1674-81 (2013).
9. Figlioli, G. *et al.* Novel genome-wide association study-based candidate loci for differentiated thyroid cancer risk. *J Clin Endocrinol Metab* **99**, E2084-92 (2014).
10. Figlioli, G. *et al.* Novel genetic variants in differentiated thyroid cancer and assessment of the cumulative risk. *Sci Rep* **5**, 8922 (2015).
11. He, H. *et al.* Genetic predisposition to papillary thyroid carcinoma: involvement of FOXE1, TSHR, and a novel lincRNA gene, PTCSC2. *J Clin Endocrinol Metab* **100**, E164-72 (2015).
12. Porcu, E. *et al.* A meta-analysis of thyroid-related traits reveals novel loci and gender-specific differences in the regulation of thyroid function. *PLoS Genet* **9**, e1003266 (2013).
13. Kucharzewski, M., Braziewicz, J., Majewska, U. & Gozdz, S. Concentration of selenium in the whole blood and the thyroid tissue of patients with various thyroid diseases. *Biol Trace Elem Res* **88**, 25-30 (2002).
14. Kim, H.Y. The methionine sulfoxide reduction system: selenium utilization and methionine sulfoxide reductase enzymes and their functions. *Antioxid Redox Signal* **19**, 958-69 (2013).
